# Constructing Research: A Theoretical Perspective on Research and Creativity in Architecture

**DOI:** 10.1177/19375867251353730

**Published:** 2025-07-07

**Authors:** Melissa Piatkowski

**Affiliations:** ^1^Centre for the Study of Professions (SPS), 60499 Oslo Metropolitan University, Oslo, Norway

**Keywords:** evidence-based design, creativity, architecture, healthcare design, research in practice

## Abstract

**Objective:** This article provides a conceptual exploration of research and creativity within the profession of architecture, including a proposed theoretical framework exemplifying research as a catalyst in the creative process in practice. **Background**: There is a growing recognition among thought leaders in the profession of architecture that both intuition and research have a rightful place in the creative process. However, there remains a pervasive concern in practice that research can threaten or detract from creativity. There is an opportunity for conceptual integration of these approaches. **Methodology**: Inspired by Reflective Equilibrium, this work is an analysis of the potential synergies between research and creativity in architecture. The dichotomy between normative assumptions of the two concepts is reassessed. **Results**: A conceptual model called The Supportive Model of Research is proposed, showing how research is more likely suited as a catalyst within the creative process in the profession of architecture. This model explores three ways research can benefit the creative process: (a) fortification; (b) protection; and (c) elevation. Three narratives are presented to illustrate the three components of the model. **Conclusions**: The apparent dichotomy between intuition and research may be false; after all, there is rarely a one-size-fits-all approach to every problem. There is a need to construct a new paradigm with clarity around the benefits of an expansive view and an integrated approach, with research as a support for the virtues of creativity. The work in this paper is an attempt to “break ground” on this conceptual construction project.

## Introduction

While creativity in architecture tends to build on imagination and possibilities, many of the daily challenges architects face are entrenched in rules, systematic processes, and fixed boundaries. So, it may be no surprise that some architects see research as yet another potential threat to creativity (Brandt et al., 2010). However, if we assume that the goal of architecture is to create something new and valuable, one must question the value of an original idea that has not been evaluated.

Proponents of research advocate for designers to use research to push their design decision-making process from the shaky grounds of intuition to a solid position of creativity rooted in evidence. This point is well illustrated by Harris and other leading evidence-based design (EBD) experts in their book *A Practitioner's Guide to Evidence-Based Design*:

Doesn’t EBD restrict creativity and innovation? No. However, EBD may restrict unfounded, novel solutions that add little value. […] When evidence about how something works—or doesn’t work—is considered a threat to creativity, it is likely that a failure of imagination is to blame, an inability to consider a wide range of relevant factors to find new ways of doing something. (Harris et al., 2008, p. 25)

Given the importance of both creativity and research in architecture, and the increasing pressure to utilize research in the field, I aim to explore how these approaches can act in concert with one another. There are many helpful process frameworks available to show how to use research in design [see, for example, The Evidence-Based Design “Wheel” (Malone et al., 2011)], and there are also frameworks that show how EBD and intuitive design relate to best practice [see, for example, Sources of Design Inspiration (Lundin, 2015) or Hamilton's discussion of the bell curve (Hamilton, 2023)]. In this article, I propose a new conceptual model, called The Supportive Model of Research in the Creative Process, as a complement to these existing resources. This model is meant to clarify and reframe the conceptual understanding of research not as a threat but rather as an integrated catalyst in the creative process in architecture.

*This model is meant to clarify and reframe the conceptual understanding of research not as a threat but rather as an integrated catalyst in the creative process in architecture.*

### Creativity as a Virtue: Originality Through Intuition, Intentionality, and Autonomy

Creativity is perceived as a central tenet in the profession of architecture (Ahuja et al., 2017; Styhre & Gluch, 2009). But what is creativity? Or perhaps the more important question is, what is it about creativity that is perceived to be at stake within the profession?

A basic definition from Oxford Languages (2023) captures the normative view that creativity is “the use of imagination or original ideas to create something; inventiveness.” Philosophical and theoretical attempts to describe creativity usually involve the following normative qualities: originality, intuition, intentionality, and autonomy, with originality at the forefront of nearly every description (Feldhusen & Goh, 1995; Gaut & Kieran, 2018). Beyond “the element of novelty” (Gaut & Kieran, 2018, p. 3), philosophers have posited that for a thing to be considered creative, it must not only be new, but it must be “interestingly new” or “surprising” (Boden, 2018; Gaut & Kieran, 2018, p. 3).

In addition to the qualities that make something creative, there are widely held beliefs in architecture about where creative work originates from. Traditionally, higher value has been placed on creative work derived from the internal intuition of the individual, as opposed to external or collaborative influences (Ahuja et al., 2017; Ese & Ese, 2022; Horn, 2019) [although this norm appears to be shifting as the profession becomes increasingly interdisciplinary (Ahuja et al., 2017)]. And many architects would agree with philosophers of creativity that truly creative work cannot be conceived through random or accidental circumstance (Gaut & Kieran, 2018; Stokes, 2014), but that it should be intentional, goal-oriented, and based in the agent's knowledge, skills, and discretion (Stokes, 2014, p. 164). In this vein, architects strive to protect their autonomy and authority in order to maintain intentionality in the creative process (Ahuja et al., 2017; Styhre & Gluch, 2009).

### Creative Virtue Under Threat?

Many traits that seem oppositional to the qualities of creativity are precisely what architects face in their day-to-day work. As architecture and design critic Oliver Wainwright writes:

It is a dense minefield of rules and regulations that governs everything from the size of windows to the pitch of rooftops, the depth of stair treads to the gradient of slopes—even where to put light switches.[…] [E]very aspect of a new building has been quantified and calibrated before the designer even sets pen to paper. (Wainwright, 2013, para. 2)

This everyday work—managing tight budgets and compressed schedules and navigating the nuanced requirements of building regulations, standards, and codes—stands in stark contrast to the values around intuition and innovation presented in architecture schools and glossy design magazines (Styhre & Gluch, 2009). Opportunities for creativity are constantly value-engineered (i.e., cut) out of the project or steamrolled by “the business” of architecture (i.e., the bottom line, “the money”).

## Research as an Enabler—Or Another Threat?

Proponents of research in architecture often encounter the concern voiced by architects that research is yet another potential threat to creativity (Hamilton, 2014). There are many potential explanations for this concern, some of which can be traced to the vast array of ideas about what *research* is in the first place. The term “research” is used to describe “both a source of knowledge and the process of creating new knowledge” (Peavey & Vander Wyst, 2017, p. 144). In other words, one can apply the results of existing research (or evidence), or one can conduct new research, but the term is often thrown around without making this critical distinction (Peavey & Vander Wyst, 2017). The term is used to describe a wide variety of functions as well, such as seeking sources of inspiration or referencing building standards. Further, there is a widespread assumption that empirical research means highly quantitative research that falls into a positivist paradigm, which may contribute to the perception that research is oppositional to the more interpretive, relative concept of creativity. And regarding the use of existing research, there is a concern that research findings are a prescription for specific, standardized solutions (Rashid, 2013; van der Zwart, 2021).

The seeming opposition between research and creativity falls within a longstanding debate about different intellectual approaches to life and society (e.g., art vs. science, scientific vs. literary, humanities vs. science, etc.). Expectations around scientific literacy vary among different professions (see, for instance, Snow, 1959), and this debate takes a distinctive form in architecture, especially in healthcare design. But must creativity and research be viewed as a dichotomy? While there are differences, there are undoubtedly many similarities. However, these perceptions persist, and I have noted some of the abstract normative principles of creativity and research in Table 1 below, highlighting the similarities (without shading) and differences (with shading) between each construct.

**Table 1.** Constructing Constructs: Assumed Oppositional Qualities of Creativity versus Research.

	Creativity	Research**^a^**
Normative qualities	Original (Feldhusen & Goh, 1995; Gaut & Kieran, 2018)	Original
	Interestingly new, imaginative, and innovative (Boden, 2018; Gaut & Kieran, 2018)	Significant, relevant (Belcher et al., 2016)
	Intentional (Gaut & Kieran, 2018)	Systematic (Leung, 2015)
	Unique, distinct	Generalizable, transferable (Leung, 2015)
	Mystical, undefinable process (Anstey, 2003; Horn, 2019; Styhre & Gluch, 2009)	Transparent process (Leung, 2015)
	Valuable (varied opinions on who determines and attribution to the process or the product)	Valuable (significant contribution to the body of knowledge)
	Focus on possibilities, plurality, *what could be*	Focus on understanding the truth, *what is* (Leung, 2015)
	Subjective (Lundin, 2015, p. 123)	Objective, rational (Lundin, 2015, p. 123)
Idealized source	Tacit knowledge (Polanyi, 1967) based on intuition and experience (Hamilton, 2023) based on innate understanding, genius (Anstey, 2003)	Rigorous process (Hamilton, 2023)
	Intuition (Ahuja et al., 2017; Ese & Ese, 2022; Horn, 2019)	Empirical process
	Autonomy (Ahuja et al., 2017; Styhre & Gluch, 2009)	Collaboration
	Innate quality (genius) (Anstey, 2003)	Acquired skill (and some genius)

^a^ It is important to emphasize that these are widely assumed qualities of research within the profession of architecture, which tend to lean toward a more positivistic, realistic, quantitative paradigm (van der Zwart, 2021). This is not meant as a summary or definition of research, which, for example, would also include research done within epistemologies like contextualism and constructivism.

*Note*. Normative differences between Creativity and research are indicated by shading, and similarities are shown without shading.

There are many different process frameworks intended to guide the application of research in the design process (e.g., design thinking, human factors/ergonomics, Lean, EBD, etc.). The variation in research tools and resources likely adds to the variation in the conceptualization of what “research” means in architecture practice; however, these various process driven approaches are all based to some extent on the scientific method, an iterative process that can be adjusted and applied in a variety of ways depending on the question or problem at hand (Gauch, 2003). The scientific method is often represented as a sequence of steps involving observation, asking a research question and/or a hypothesis, and gathering data to evaluate the hypothesis. “Research” in the scientific method may only refer to the use of existing (secondary) data, or it may also involve gathering new (primary) data.

In this article, I will focus on one of the most well-known research approaches in architecture: EBD. The Center for Health Design introduced the EBD process in the early 2000s to the architecture industry as a way for design practitioners to integrate the scientific method into the traditional design process (About EBD: What Is Evidence-Based Design (EBD)?, 2008). The concept was modeled after evidence-based medicine (EBM) (Stichler & Hamilton, 2008), and defined by The Center as “the process of basing decisions about the built environment on credible research to achieve the best possible outcomes” (About EBD: What Is Evidence-Based Design (EBD)?, 2008). This definition alone leaves the concept of “credible research” open to interpretation, but The Center's educational materials expand on the definition by emphasizing the importance of using the best information (or evidence) available in cases where there is no credible (or relevant) research on a particular design topic (The Center for Health Design, 2022). And while The Center tends to use the term “evidence-based design” to refer to research or evidence that precedes and informs design decisions, there may be some additional confusion caused by the use of the term when used to reference the process of research done during or after design for the sake of design evaluation [e.g., post-occupancy evaluation (POE)]—meaning the design itself may not be evidence-*based*.

When considering how research is conceptualized and understood in architecture, it may be helpful to consider how EBD's parent concept, EBM, is characterized. The most cited definition reads, “The practice of evidence-based practice means integrating individual clinical experience with the best available external evidence from systematic research” (Sackett et al., 1996, p. 71). EBM is discussed as a science, as an art, and as a craft (Eriksen, 2022), and yet, there is a tendency among architects to think of EBM within a positivistic paradigm (comprised of qualities such as those outlined in the Research column in Table 1). For instance, in his reflections on the *4th annual Architecture Research Care & Health (ARCH19) conference*, Johan van der Zwart comments:

Evidence-based medicine rests on a specific set of philosophical assumptions about research and what is regarded as best evidence […] a system of beliefs, values, and ideas that define what is regarded as legitimate contributions to a field of research—is a positivistic approach that views the world as an observable reality in which evidence is based on facts that can be objectively measured and described. (van der Zwart, 2021, pp. 12–13)

The derivation of EBD from EBM is noteworthy because it may explain why it seems well-suited to (and perhaps pigeonholed within) the field of healthcare design (Cama, 2009; Shepley & Watson, 2013). It may also explain why quantitative research is perceived to hold a higher value than qualitative research in the field (Rashid, 2013; van der Zwart, 2021), although, to my knowledge, this stance has not been explicitly stated by proponents of EBD.

**Figure 1.**
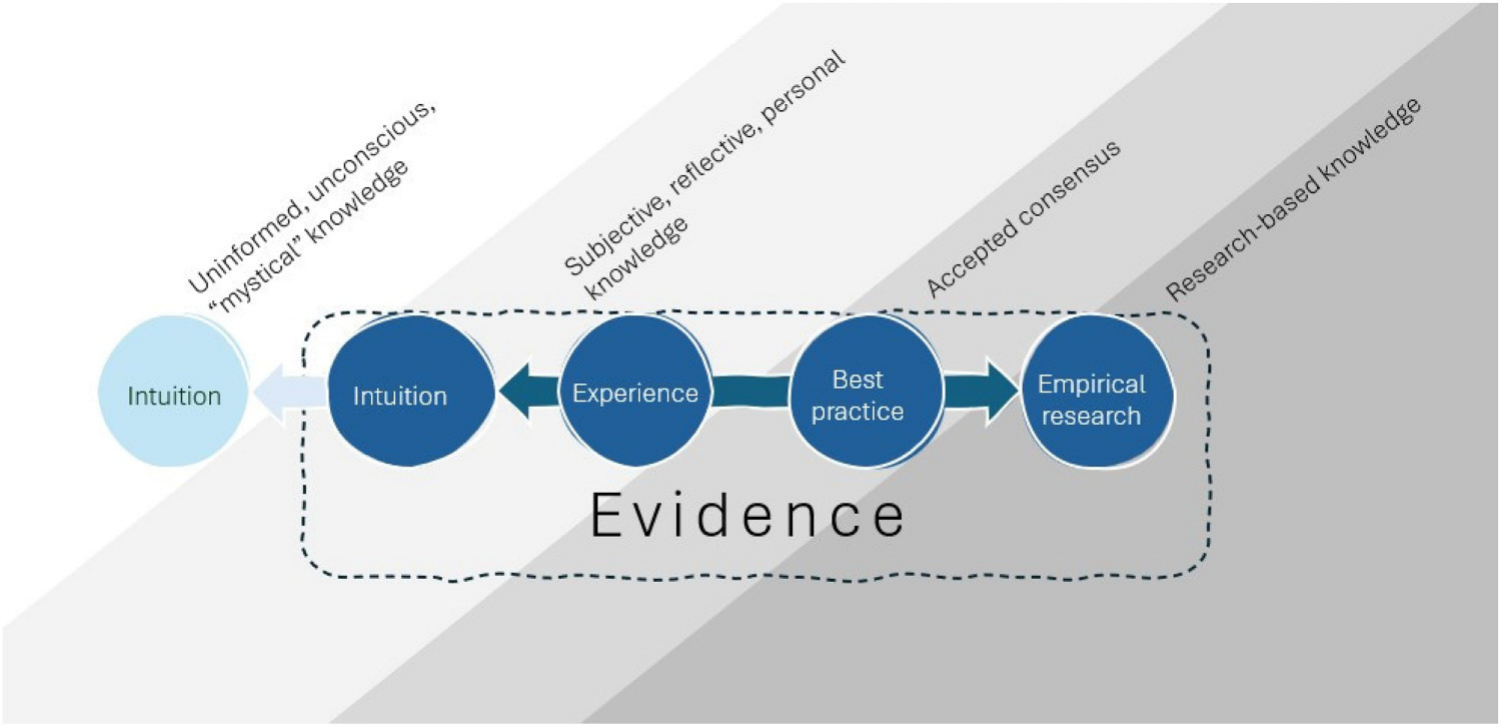
Spectrum of (The Construct of) Evidence.

*Note*. Informed by “Diagram of The Principal Forms of Knowledge Involved in The Architectural Profession” (p. 93), by Lundin (2015), PhD Thesis, Chalmers University.

Proponents of EBD tend to advocate for the conceptualization of “evidence” on a spectrum of different types of knowledge, with intuitive knowledge as one extreme and empirical research-based information as the other (Lundin, 2015). This spectrum is illustrated below in Figure 1. This discussion around intuitive knowledge can be somewhat confusing, which may contribute to misconceptions and debate. There are numerous and varied accounts of intuition as it relates to science, including intuition as a powerful opposing force to scientific ideas (e.g., Shtulman, 2022), intuition as integral to scientific reasoning (e.g., Osbeck, 2018), and intuition as a valid source of knowledge and ideas which can be tested using scientific methods (e.g., Hamilton, 2019). However, the meaning is generally conceptualized in one of two ways. One belief is that intuition is an outcome derived from reason and experiences (indicated by darker shading; intuition that falls within the boundaries of evidence), and another belief is that intuition is a process that results in a sort of mystical inner-knowing “without awareness of the process itself” (indicated by lighter shading) (Järvilehto, 2015, p. 6). The latter may be more in line with the traditional view in architecture regarding the ideal source of creativity (described earlier as potentially oppositional to research), and the former is more in line with the view touted by advocates of EBD, that experience is a type of evidence. A deeper discussion around what intuition is—or the cognitive, conscious, or unconscious processes of reasoning behind it—is beyond the scope of this paper. The intention here, instead, is to focus on general beliefs and perceptions of the concept in terms of architectural creativity. Suffice it to say, “[t]here is a perfectly good ordinary sense of ‘intuition’ in which what is meant is judgements for which we may not be able to produce explicit backing or justification” (Annas, 2011, p. 171, footnote 1).

The debate between advocates of research/EBD and creativity/intuition became most heated in the early 2000s in the healthcare design sector within the context of a larger discussion around the potential role of the built environment in adverse safety events (Rubin, 1998). However, in recent years, some of the most devoted advocates of each dogma are considering a more expansive view. For instance, through ongoing debate and dialogue, two prominent thought leaders in the field, Kirk Hamilton, Professor Emeritus at Texas A&M University College of Architecture (who coined the term “evidence-based design”), and Stefan Lundin, architect and partner at White Arkitekter, have come quite close to a consensus on the topic (Table 2).

**Table 2.** Intuition versus Research: From Polarization to Consensus Among Thought Leaders.

Approximate Timeline	Advocates of an Evidence-Based Approach	Advocates of an Intuition and Creativity-Based Approach
2000		
	“An architect has an obligation […] to use the most reliable information available.” (Hamilton, 2004, p. 2)	
		“Evidence Based Design represents an attempt to ‘medicalize’ architecture.” (Wagenaar, 2003, p. 9)
	Research does not restrict innovation; it restricts “unfounded novel solutions that add little value.” (Harris et al., 2008, p. 25)	
2010		
		“Some architects fear that the use of evidence-based design may inhibit creativity through prescriptive rules and prescriptive solutions.” (Brandt et al., 2010, Interview with Vivian Loftness)
	“EBD is not about rigid rules. The same evidence can help designers create quite different approaches for addressing similar issues or objectives.” Roger Ulrich (Marberry, 2010, para 73)	
		“Design is not science; design is creativity.” (Millard, 2012, para 15)
	“Much that we design must inevitably be generated from a creative, intuitive decision, the result of which the architect simply believes will be worthwhile. I suggest the obligation for an evidence-based practitioner is to deliberately state the design hypothesis embedded within the intuitive decision, and to carefully measure the related outcomes.” (Hamilton, 2014, p. 140)	→
2015		
	←	“Intuition goes beyond evidence. It is a tool for progress, a way toward future knowledge and increased evidence. Intuition is, in my world, almost the same as the requested hypothesis. I think to disregard your intuition is unscientific!” Stefan Lundin (2015, p. 125)
2020		
	“Instead of using evidence as a base for design, it should be regarded as an increased amount of information that enriches design.” Stefan Lundin (Lundin, 2021)	
	“Intuition is not necessarily the absence of knowledge. It's not random; it's not irresponsible. And it is ultimately fascinating how often intuitive decisions turn out to be supported by evidence.” Kirk Hamilton (The Center for Health Design, 2021)	

In Table 2, note the shifting of boundaries between proponents of each approach (represented by borders in the table) toward consensus. While these quotes are mere snippets of a much larger and more complex dialogue, they illustrate the progression of ideas in the conversation over the years.

## Methodology

The exploration of research and creativity in this article is inspired by the idea of “reflective equilibrium” (Rawls, 1971). As described by Thacher (2006):

[t]he method of reflective equilibrium rests on the idea that we try to criticize, clarify, and improve our existing views about normative ideals by reflecting on the implications they have for other convictions. In the process, we try to bring everything into harmony by modifying convictions that come to seem misguided once we have examined them in the light of other commitments. (p. 1647)

This quote describes the horizontal dimension of reflective equilibrium, focused on the harmonization of normative concepts. Reflective equilibrium also has a vertical dimension, which references empirical information to expand on and challenge the theoretical aspect. This iterative approach provides a new point of departure in the conversation around the concept of research in architecture—which may allow for a more interpretive paradigm—one that may better align with the tradition of creativity.

The model proposed in this article has been developed in two phases: first, by examining multiple theories on *creativity* as a virtue within the profession of architecture, based mainly on the most current philosophical debate compiled in the book *Creativity and Philosophy,* edited by Gaut and Kieran (2018). The second phase involves reflection on the concept of *research* and the inherent opportunities to fortify, protect, and enhance what (virtue) is at stake in the creative process. This second phase includes an exploration of research-creativity alignment and how this looks in practice through three narratives.

The three narratives presented are comprised of patterns of circumstances from projects I have worked on or been acquainted with through my experience as a researcher in the field of healthcare architecture and design. These are composites based on my reflections on architecture projects where research was integrated into the creative design process.

## The Supportive Model of Research in the Creative Process: Three Narratives

**Figure 2.**
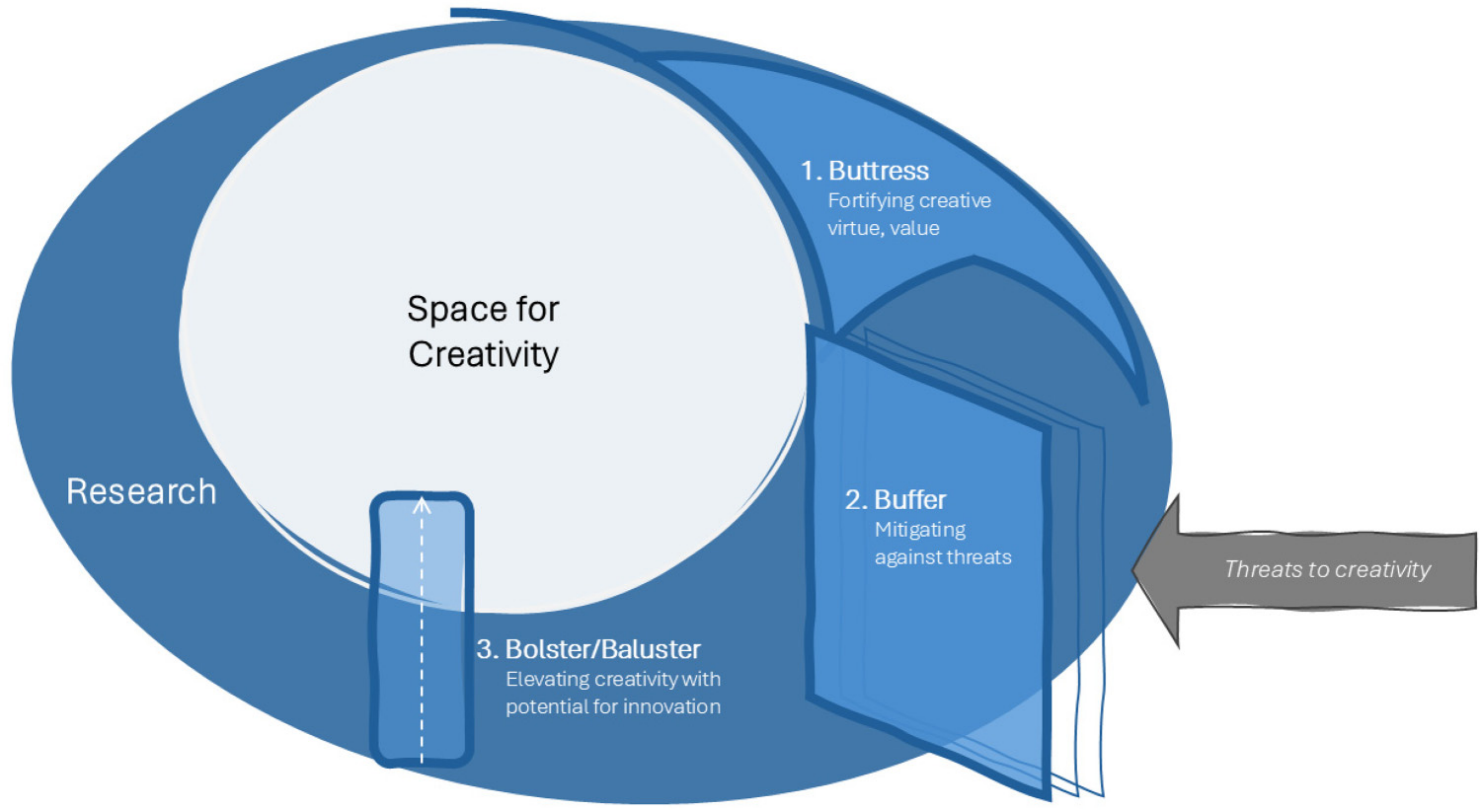
The Supportive Model of Research in The Creative Process.

The following section explores three ways research can benefit the creative process. These views are presented as three architectural elements (Figure2):

1. Research as a buttress: Fortification of creative virtue;
2. Research as a buffer: Protection and mitigation against threats to creativity;
3. Research as a bolster/baluster: Elevating creative quality and potential for innovation.

This conceptual model shows the space for creativity at the center, surrounded by a supportive layer of research, with challenges encroaching in from the outside. The research buttress, buffer, and bolster each provide a different type of synergistic support to the creative process. In the following section, I describe the meaning of each relation between research and creativity through three narratives. These narratives come from scenarios in healthcare architecture and design, but the concepts have relevance in any design sector.

### Research as a Buttress: Fortification of Creative Virtue

There are many examples of architectural projects designed through creative intuition that have been deemed successful. But who or what determines success? If we return to the qualities of creativity in Table 1, we see that the virtue of creativity involves *value*. The success of a creative process begins with a question about how we conceptualize *value* within the construct of creative virtue. The word *value* is often conflated with the word *valuing* (Audi, 2018). “*Value* as a kind of worth is one thing; *valuing*, as something close to caring, is quite different… Valuing is not always directed toward the valuable” (Audi, 2018, p. 34). The strength of creative virtue is on somewhat shaky grounds without *buttressing* or *fortification* of value.

*The strength of creative virtue is on somewhat shaky grounds without *buttressing* or *fortification* of value.*

One means of confirming *value* is through e*valu*ation. While research is not a common part of the traditional curriculum in architecture schools, many architects are familiar with POE. Gathering data on outcomes of interest through the POE process is essentially a means to test an architect's hypothesis about their creative design concept. Was it effective?

Consider, for instance, a scenario where a firm is hired to design an eye surgery center. With creative intention focused on designing something original, the architect seeks inspiration from the human eye and proposes a design parti (i.e., the central driving concept) based on the anatomy of the eye. Her concept includes an extensive curtain wall of windows inspired by the cornea, which will allow sunlight to stream into the interior of the building. Intuitively, she believes that natural light will enhance the esthetic qualities of the space. The conceptual design is approved and is eventually built.

**Figure 3.**
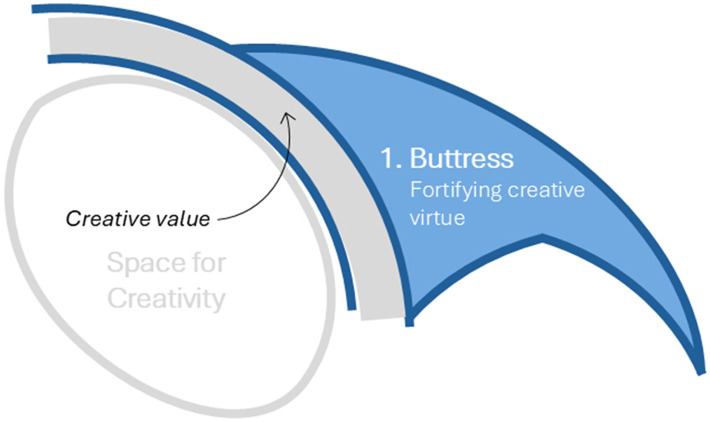
Research as a Buttress: Fortification of Creative Virtue.

Several years later, the same firm is hired again to handle a renovation project. This time, they decide to conduct a small POE to understand if the original design intent was achieved, and to inform the new design. They ask: How has the design influenced the quality of experience for patients and staff? The design team gathers feedback through interviews with staff and learns that while they have been generally happy with the design, the intense sunlight streaming through the windows into the waiting room has been a major source of discomfort for visually impaired patients and patients whose eyes have been dilated. Taking this evidence into account, while preserving the creativity of the original design parti, they determine that the exterior windows are more appropriate for staff who can enjoy the sunlight and views, and they propose relocating the waiting room to the center of the building, where the interior lighting has been selected to optimize visual comfort for patients (Figure 3).

In this example, we see how research can be a differentiator between a mere original design that the architect *values* and a creative design that has *value* (whether it be to the users, to other architects, to commissioners, or to other stakeholders). The eye surgery center scenario is an example of a minimal application of new research with an important difference in creative virtue. A more rigorous approach with additional research methods (e.g., literature review, observation, surveys) could deepen the architect's understanding of the user experience and inspire ideas around creative priorities—and may have the potential to further strengthen the value of a creative notion.

*research can be a differentiator between a mere original design that the architect *values* and a creative design that has *value*.*

### Research as a Buffer: Protection and Mitigation Against Threats

The driving force behind many projects in the current economic climate is not a central concept or vision but simply the budget and the schedule. These forces will limit the space and opportunity for creativity. Research may act as a *buffer* against the daily challenges and threats that architects face, a means to protect an architect's autonomy and jurisdiction over the space for creativity. For instance, research can help architects make a business case to justify and argue for creative design ideas (Hamilton, 2020b; Sadler et al., 2011). Consider a case where an architect proposes a design concept based on their intuition that art will improve the patient experience in a psychiatric hospital. The client agrees that having art would be nice. But art is expensive, so is it necessary compared to other priorities in the budget? It is easy to imagine that the architect's proposal (if considered at all) would be value-engineered out of the final budget.

**Figure 4.**
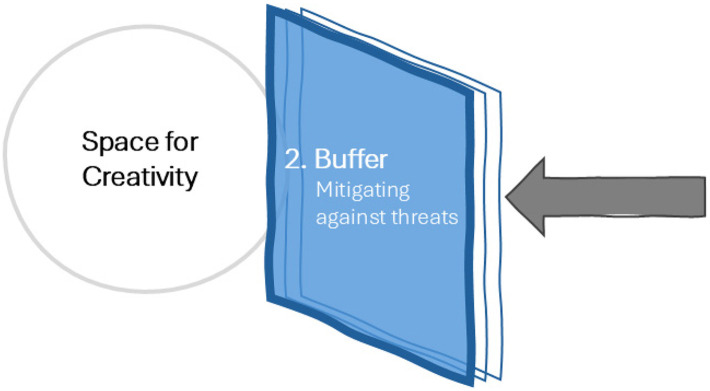
Research as a Buffer: Protection and Mitigation Against Threats.

But imagine that the architect refers to empirical research on the impact of art in psychiatric facilities during the design process and as part of their argumentation. For instance, they might find a study conducted by Nanda et al. (2011), where researchers compared the impact of rooms with art to rooms without art on patient agitation and anxiety levels, as well as anxiety medication dispensed. Findings in this study showed that patients in the room with art, especially art depicting nature, experienced lower anxiety and agitation and required less anxiety medication than those in the room with no art. The potential annual cost savings in terms of time and money for medication totaled nearly $30,000 USD (Figure 4).

In this scenario, the architect could walk into the next client meeting armed with evidence to make a compelling business case for investment in his intuition-based creative idea. Instead of creating a new threat, research can reduce the impact of the real budgetary threat. Instead of relinquishing authority, the architect is far more likely to maintain autonomy over the creative process here *because of* the research they have used to justify it.

*Instead of relinquishing authority, the architect is far more likely to maintain autonomy over the creative process here *because of* the research they have used to justify it.*

### Research as a Bolster: Elevating Creative Quality and Potential for Innovation

**Figure 5.**
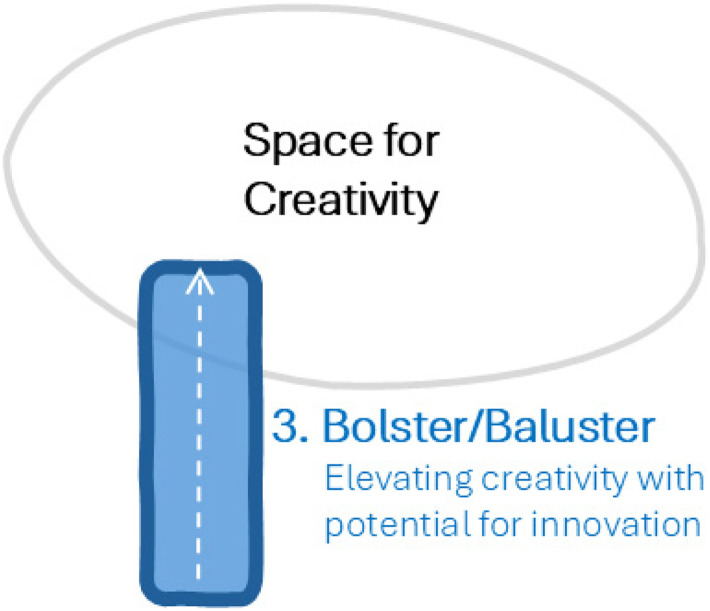
Research as a Bolster: Elevating Creative Potential.

In addition to fortifying and protecting creativity, research can provide a pathway to exploring and understanding a problem. Through collaborative problem exploration, research can inform priorities and reveal opportunities for innovation (Figure 5).

The following narrative comes from Yale New Haven Hospital in the USA, designed by Salvatore Associates and CAMA Design (Cama, 2009). This design team used an EBD process, which began by creating an interdisciplinary design team, including care providers. Clinic leadership was focused on specific care priorities, including hand hygiene and patient-provider communication. The design team read studies indicating a link between sink location and visibility in the exam room and hand hygiene compliance. There is very little space in a typical exam room, and the sink is often directly in front of the provider when they enter the room, so the designers asked the care providers what else might deter them from handwashing. The providers explained that when they wash their hands, they are forced to turn away from the patient, which detracts from the patient's communication and overall experience.

**Figure 6.**
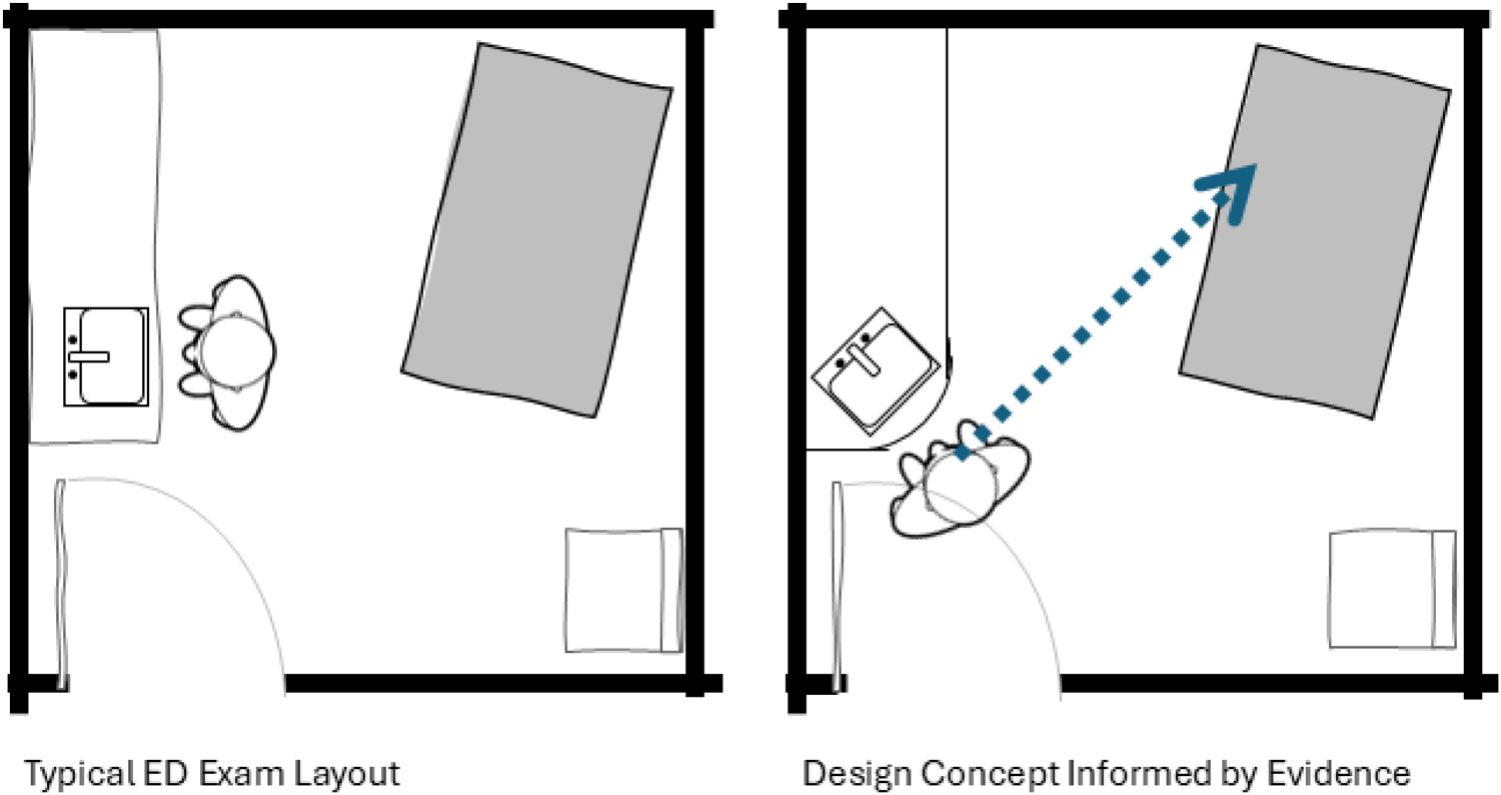
Example of a Small Creative Innovation With Significant Creative Value.

*Note*. The floorplan sketch on the right is based on Yale-New Haven Hospital Children's Hospital Emergency Department (Cama, 2009).

At this early stage in the design process, the design team used research as a jumping-off point to push toward something completely new. In addition to reading the existing research, they gathered further evidence through interdisciplinary collaboration. They then developed a design hypothesis that reorientating the sink could allow the care providers to wash their hands while maintaining eye contact. This led to a novel 45-degree positioning of the sink to support both care priorities (see Figure 6).

In this case, research helped the design team to alternate between creativity and research, to better understand the problem and previously documented potential solutions, arriving at a new, innovative design strategy. The difference between research as a prescription and research as information is the key to creativity in the “bolster” concept. Using research findings as a directive to “copy and paste” would clearly not support creativity and, further, would rarely be appropriate or relevant due to the major variation across projects. Instead, the bolster concept is centered on taking research findings as one element of information (or inspiration) within a larger context, which then requires creativity to adapt design solutions in appropriate ways to specific contexts. Perhaps the level of innovation in the Yale New Haven example would not be considered earth-shattering, yet the intentionality of the creative process has resulted in something that would appear to hold significant creative value for the users. Research may provide the discretionary space that can elevate basic creativity and problem-solving to a higher level, toward innovation.

*Research may provide the discretionary space that can elevate basic creativity and problem-solving to a higher level, toward innovation.*

## Discussion

While the EBD approach has become increasingly well-known in practice, research in most architecture education remains limited (Hamilton, 2020a). Architectural education has a long tradition of learning to design by doing, by making, and by creating in the design studio—rather than by reading or researching (Shilling, 2024). And for professionals interested in using research, it is incredibly challenging to know where to start, how to access the “right” information, and how to make sense of dense scientific literature. While there is room for growth regarding research literacy, architects do not need to change careers and become researchers before using research toward creativity. There are many opportunities for practitioners to become informed consumers of research and to engage in the research process. For instance, there are many open-source tools and resources to support an EBD process available through The Center for Health Design (e.g., The EDAC program, the Knowledge Repository, and several Interactive Design Diagrams) (The Center for Health Design, 2025).

The model proposed in this article is weighted toward the benefits of research for creativity. While emphasizing research as a positive influence is intended to push the boundaries of how research is conceptualized in the profession, the goal is a balanced and integrated approach. Just as creativity can benefit from research, research can benefit from creativity, too. This may be especially pertinent in healthcare design, where tradeoffs or “wicked problems” (Rittel & Webber, 1973) abound. Research can support the creative process, but the research itself must also be approached through the lens of creative thinking. Every project is unique, and the body of empirical research in healthcare design is by no means comprehensive or relevant to every design problem. Creative intuition guided by experience is and will likely always be essential to the architectural design process.

### Limitations

It is important to recognize that the representations of *creativity* and *research* in the background section of this article are based on many commonly held assumptions within the profession. However, there is a lack of clarity, even among experts, around what these terms mean. The discussion around these concepts may appear too reductive and lacking nuance, but the simplification can hopefully spark discussion and provide a theoretical jumping-off point for future work.

While writing the narrative examples in this article, I was struck by the lack of documented or empirically evaluated accounts of specific process-led approaches in the design process. As I reflect on my experiences with research and EBD, I know that when these processes are examined, architecture firms tend to keep the results in-house as proprietary knowledge. Therefore, I have provided narratives based on typical experiences that I hope readers will find relatable, useful, and as a starting point for further dialogue.

There is perhaps a more profound question in the context of healthcare design behind “Is research a threat to creativity?”—and that is: “Has EBD actually improved healthcare?” While answering this question is beyond the scope of the current paper, it is an important consideration. There is a great need for research that evaluates the impact of EBD and other research approaches on the field and in the human experiences of these designed environments. However, even if it is determined that EBD has not made a difference in healthcare, the default option is creativity. And the proposed model shows that, perhaps, research can protect the sphere of creativity.

## Conclusion

When considering the influence of scientific research on creativity, there is often a focus on the differences between these two forces, but it may be helpful instead to explore the commonalities. Creativity and research are both types of information. Creativity and research are both paths to decision-making, justification, and problem-solving (Polanyi, 1967). Despite the obsession with novelty in both creativity and in research, some of the most incredible innovations in both areas begin through imitation, inspiration, and influence from previous work. Scientific discoveries often require creative thinking to develop hypotheses and experimentation methods. Creativity is essential when “translating” research into practice. Creativity and scientific thinking are both considered important intellectual virtues (Baehr, 2018), and each of these can be beneficial within both the arts and sciences. Architects are not faced with a binary choice between research and creativity—but instead have access to two sides of the same coin—if the coin is problem-solving.

*Architects are not faced with a binary choice between research and creativity—but instead have access to two sides of the same coin—if the coin is problem-solving.*

The proposed framework is a working model, a hypothesis if you will, and it should be tested. The examples are quite simplistic, and the value of the model would benefit from consideration in more complex cases, and in sectors beyond healthcare. There is a need for *research on* EBD and research-informed design—to better understand how these process-led approaches influence the design process and elements of design like creativity. The work presented in this article is part of a larger research project that aims to address this need, which includes a qualitative exploration of how architects perceive research, EBD, and creativity. I invite other researchers to focus specifically on creativity as an outcome when evaluating the effectiveness of different process-led approaches and to explore the proposed model within and beyond the healthcare design sector.

The Supportive Model of Research in the Creative Process is intended to provide conceptual clarity around the emerging consensus on the complementary qualities of research and creative intuition. Through the buttress, the buffer, and the bolster, we can envision the synergy between these two different and interrelated concepts when operating in a much larger context.

## Implications for Practice

- Promote understanding of the relationship between research and creativity within the profession of architecture.
- Clarify the emerging consensus on the complementary and mutually beneficial qualities of research and creative intuition.
- Propose a conceptual model to facilitate an understanding of the beneficial role of research in the creative design process.
- Provide a clear visual representation of how research can help to strengthen, protect, and enrich creativity.
